# The making of nursing practice Law in Lebanon: a policy analysis case study

**DOI:** 10.1186/1478-4505-12-52

**Published:** 2014-09-05

**Authors:** Fadi El-Jardali, Rawan Hammoud, Lina Younan, Helen Samaha Nuwayhid, Nadine Abdallah, Mohammad Alameddine, Lama Bou-Karroum, Lana Salman

**Affiliations:** Department of Health Management and Policy, American University of Beirut, Riad El Solh, PO Box 11-0236, Beirut, 1107 2020 Lebanon; Knowledge to Policy (K2P) Center, Faculty of Health Sciences, American University of Beirut, Riad El Solh, Beirut, 1107 2020 Lebanon; Center for Systematic Reviews of Health Policy and Systems Research (SPARK), American University of Beirut, PO Box 11-0236, Riad El Solh, Beirut, 1107 2020 Lebanon; Research, Advocacy and Public Policy-making, Issam Fares Institute for Public Policy and International Affairs, American University of Beirut, Riad El Solh, PO Box 11-0236, Beirut, 1107 2020 Lebanon; Department of Clinical Epidemiology and Biostatistics, McMaster University, CRL-209, 1280 Main St. West, Hamilton, L8S 4 K1 ON Canada; Rafic Hariri School of Nursing, American University of Beirut, Riad El Solh, Beirut, 1107 2020 Lebanon; Order of Nurses in Lebanon, Sin El-Fil, Beirut Hall Street, Chaoul Center, PO Box 55311, Beirut, Lebanon; University of California, Berkeley, 228 Wurster Hall #1850, Berkeley, CA 94720-1850 USA

**Keywords:** Evidence-informed policymaking, Lebanon, Nursing law, Policy analysis

## Abstract

**Background:**

Evidence-informed decisions can strengthen health systems, improve health, and reduce health inequities. Despite the Beijing, Montreux, and Bamako calls for action, literature shows that research evidence is underemployed in policymaking, especially in the East Mediterranean region (EMR). Selecting the draft nursing practice law as a case study, this policy analysis exercise aims at generating in-depth insights on the public policymaking process, identifying the factors that influence policymaking and assessing to what extent evidence is used in this process.

**Methods:**

This study utilized a qualitative research design using a case study approach and was conducted in two phases: data collection and analysis, and validation. In the first phase, data was collected through key informant interviews that covered 17 stakeholders. In the second phase, a panel discussion was organized to validate the findings, identify any gaps, and gain insights and feedback of the panelists. Thematic analysis was conducted and guided by the Walt & Gilson’s “Policy Triangle Framework” as themes were categorized into content, actors, process, and context.

**Results:**

Findings shed light on the complex nature of health policymaking and the unstructured approach of decision making. This study uncovered the barriers that hindered the progress of the draft nursing law and the main barriers against the use of evidence in policymaking. Findings also uncovered the risk involved in the use of international recommendations without the involvement of stakeholders and without accounting for contextual factors and implementation barriers. Findings were interpreted within the context of the Lebanese political environment and the power play between stakeholders, taking into account equity considerations.

**Conclusions:**

This policy analysis exercise presents findings that are helpful for policymakers and all other stakeholders and can feed into revising the draft nursing law to reach an effective alternative that is feasible in Lebanon. Our findings are relevant in local and regional context as policymakers and other stakeholders can benefit from this experience when drafting laws and at the global context, as international organizations can consider this case study when developing global guidance and recommendations.

## Background

Evidence-informed decisions can strengthen health systems, improve health, and reduce health inequities [1]. The Beijing, Montreux, and Bamako calls for action have emphasized the need for national governments to promote and finance Knowledge Translation towards the application of evidence-informed policymaking by developing trust between researchers, practitioners, and policymakers [2, 3]. Despite these calls, literature shows that research evidence is underemployed in policymaking, especially in the East Mediterranean region (EMR) [4–6].

In line with the World Health Organization (WHO) recommendations, a recent priority setting exercise in the EMR revealed that one of the key health systems priority concerns is related to shortages in human resources for health (HRH) particularly in the nursing workforce [7, 8]. Nursing shortages occur at multiple levels and include quantity (adequate numbers) and quality (good qualifications and clear scope of practice) [9]. The mounting complexities in patient care and acuity have increased the need for a qualified nursing workforce with consistent and coherent educational standards and policies [10–12].

The WHO, the International Council of Nurses (ICN), and the EMR Advisory Panel on Nursing have outlined recommendations for global nursing educational standards and competencies to standardize and enhance the quality of nursing care [13]. However, complying with these standards in Lebanon would require changes in legislation concerning nursing scope of practice, entry requirements, and education. These international reports triggered the initiation of the draft nursing practice law in Lebanon. This draft law has been under study by legislative authorities in Lebanon for over 12 years, and has yet to be passed.

To gain insight into the policymaking process, including the use of evidence, we have selected the draft nursing practice law in Lebanon as a case study for a policy analysis exercise. The draft nursing law was selected due to its national and regional importance and relevance, its recognition as a policy challenge, and the presence of an opportunity for change [14–16]. This case study is an example of a stagnated policy development process that aims to demystify the process of policymaking in a developing country like Lebanon and to demonstrate the complex interaction of local and international stakeholders. This policy analysis exercise aims at generating in-depth insights on the public policymaking process, identifying the factors that influence policymaking, and assessing to what extent evidence is used in this process. Selecting the draft nursing practice law as a case study, this policy analysis explores how and why this policy was developed, draws on lessons learned for informing future public policymaking, and provides insights for structuring the decision making process and integrating the systematic use of evidence [17].

### Case study background

#### Lebanese political system

The Lebanese political system is a parliamentary democratic system consisting of three powers: legislative power (represented by the Parliament elected from the people), executive power, and judicial power. Public policies can take the form of laws stemming from the legislative or decrees that implement the laws developed by executive authorities [18]. The political system in Lebanon institutionalizes the deeply rooted political sectarianism, as top government positions and seats of the parliament are earmarked by sect [19–21]. Since 2005, there has been an increased polarization in the Lebanese community. Instability in Lebanon has intensified due to political strife, security threats, and most recently, due to the impact of the Syrian crisis and the constant influx of refugees. This has led to the paralysis of the work of the government, the parliament, and, as a consequence, the policy development process in Lebanon.

#### Lebanese healthcare system

The Lebanese healthcare system is characterized as pluralistic and fragmented due to the heavy involvement of the private sector in the delivery and financing of care [22]. The Ministry of Public Health (MOPH) finances the coverage of 43% of the Lebanese population who do not benefit from another insurance plan through budgetary allocation [23]. The rest of the population are covered by various financial schemes including six different publicly managed employment-based social insurance funds, the largest of which is the National Social Security Fund that covers 23% of the population and is mandatory for the formal (public and private) sector. Other schemes include private insurance and mutual funds [23]. Despite these various financial schemes, out of pocket expenditure on healthcare remains alarmingly high at 44% [23]. Provision of healthcare services is highly privatized. Regarding primary healthcare, out of 800 facilities in Lebanon, 186 primary healthcare centers belong to the primary healthcare network which is supported by the MOPH and delivers a basic package of health services to the population across Lebanon [23]. Over half of the primary healthcare centers are owned by non-governmental organizations (51%), while the rest are owned by the MOPH, the Ministry of Social Affairs, and the municipalities [23]. As for hospitalization, 80% of hospitals are owned by the private sector and the MOPH contracts out services from these hospitals to cover its patients [23].

#### Human Resources for Health

Regarding HRH, in Lebanon there is an oversupply of physicians and an undersupply of nurses. The ratio of physicians to population is 248 per 100,000, which is the highest in the EMR and close to figures reported in the United States and Organization for Economic Co-operation and Development countries [24]. The opposite case is found with nurses as the nurse density in Lebanon is 1.18 per 1,000 individuals compared with a global average of 4.06 per 1,000 [25]. Lebanon has the 8^th^ lowest nursing density in the EMR [26]. The physician density in Lebanon is twice the nurse density [8]. Furthermore, there is a geographical maldistribution of nurses as the majority work in urban areas like Mount Lebanon (34%) and Beirut (27%), making the shortage more pronounced in smaller villages and towns particularly in rural areas [27, 28]. According to the records of the Order of Nurses in Lebanon (ONL), there are approximately 11,621 registered in the ONL [29]. However, those numbers might increase as a result of the recent work that the ONL is conducting to update their database for registered nurses. Rough estimates imply that, in order to satisfy the current need for nurses in Lebanon, the nursing workforce would need a three-fold increase [29]. Other issues affecting the nursing workforce includes high migration rates (particularly for registered nurses), low retention rates, and tough working conditions [30, 31].

As for nursing education, there are many obstacles, including different levels of entry into the career, lack of a clear-cut scope of practice, and various authorities regulating education and practice [10]. In Lebanon, nursing is taught in universities allowing students to attain a Bachelor’s degree (BS) in nursing, and in technical vocational schools, allowing students to attain a vocational degree in nursing (Baccalaureate Technique (BT), a three year program) and a higher vocational degree (Technique Superior (TS), a three year program after the BT). At the moment, less than half (47%) of the nurses in Lebanon have university BS degrees whereas the rest (53%) are vocational school graduates (33% having a TS and 20% having a BT) [25]. Currently, there are 106 vocational schools and 19 universities that teach nursing in Lebanon (excluding the number of branches) [27]. Most universities are located in urban regions (68%), while most vocational schools are located in rural regions (82%) [32]. To this day, Lebanon lacks an integrated national system responsible for the monitoring, certification, and accreditation of nursing education [29].

### The historical progress of the draft nursing practice law in Lebanon

**Figure 1:**
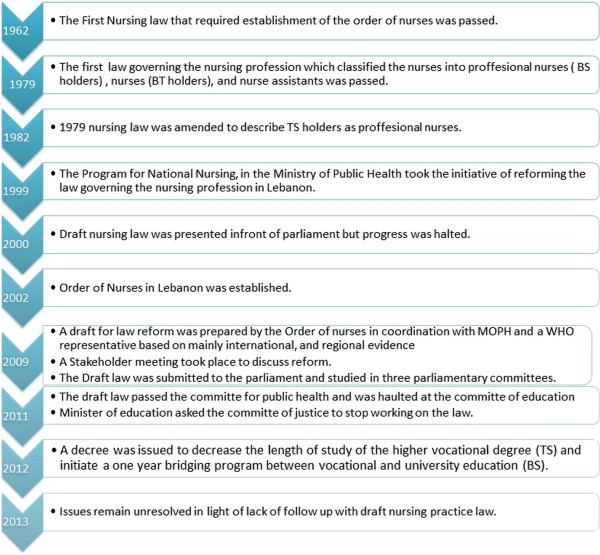
**Historical progress of the draft nursing practice law in Lebanon.**

The first nursing law in Lebanon was introduced in 1962 (Decree 9829) and the regulation of the nursing profession was within the jurisdiction of the MOPH. In 1979, the MOPH updated the law governing the nursing profession (Decree 1655) to classify and define the role and scope of nursing professionals at different levels and in 1982 it was slightly amended to become as follows [33]: professional nurse (with a BS or TS), nurse (with a BT), and assistant nurse (with a 1- to 2-year training program BP). Since 1999, there have been efforts to improve the laws governing the nursing profession in Lebanon in hopes of reorganizing and modernizing it. This resulted in a draft law called “Nursing Profession Practice Law”, which sought to replace the current law governing the nursing profession in Lebanon, Decree 1655, which was adopted in 1979. This draft law aimed to organize and enhance the nursing profession by upgrading and standardizing the entry requirements into the nursing profession and changing the nursing levels. These changes would then render the nursing workforce in Lebanon compliant with WHO and ICN standards. This draft has been under development and study for around 13 years, and to this day, these issues remain unresolved and the draft nursing law has yet to be passed. Figure 1 presents the chronological progress of the draft nursing practice law.

This policy analysis exercise aims at generating in-depth insights on the public policymaking process, identifying the factors that influence policymaking, and assessing to what extent evidence is used in this process.

## Methods

This study utilized a qualitative research design using a case study approach and was conducted in two phases: data collection and analysis, and validation. The study took place from March 2011 until June 2013. This study was both retrospective and concurrent as it evaluated the policy development process over a long period of time (13 years). At the same time, this study sought to support future policymaking and changes through this policy analysis as the draft law has yet to be passed [34].

In the first phase, data was collected through key informant interviews that covered 17 stakeholders, including two members of the parliament, two ministers, four deans of schools of nursing across Lebanon, order and syndicate representatives, and other key policymakers. Interview questions aimed at providing insight into the policymaking process of the draft nursing practice law concerning the role of stakeholders and policymakers, the context in which this draft law was developed, and to which extent evidence was utilized in the policymaking process. Key informants were purposively selected and a snowballing technique was employed to ensure that other stakeholders involved in the policy were also included. Face-to-face semi-structured interviews were conducted, lasting between 45 to 60 minutes. Interviews were digitally recorded after obtaining signed informed consent from interviewees; only four interviewees refused to be audiotaped, and their responses were then recorded by extensive note taking. The semi-structured interview tool was developed based on literature reviews and pilot tested before initiating the study. The recorded interviews were transcribed verbatim. Arabic interviews were translated into English and then back-translated to Arabic to ensure accuracy of translation. Interview transcripts were reviewed independently by two members of the research team and consequently coded. Disagreements were resolved either by consensus or discussions with the principal investigator until consensus was reached.

In the second phase, a panel discussion was organized at the ONL to validate the findings, identify any gaps and gain insights and feedback of the panelists. The panel discussion involved 12 participants including representatives from the ONL and experts in nursing administration and academia. Some of these participants had been involved in the interview phase of the study. Findings were presented to participants for discussion. Panelists shared their experience on the development of the draft nursing practice law, proposed possible next steps regarding its development, and discussed the main challenges in the policymaking process in Lebanon. Participants validated the information regarding the policymaking process that this draft law had undergone and highlighted specific barriers for its development. The panel discussion, which lasted for two hours, was recorded by note taking.

Thematic analysis was conducted. The Walt & Gilson’s “Policy Triangle Framework” provided guidance as themes were categorized into content, context, actors, and process. This framework facilitates the analysis of the content of the policy, the actors involved in the decision-making, the process by which the policy was initiated, formulated, and communicated, and the contextual factors that influenced the policy. This framework is the only framework grounded in political science, which is the science most directly focused on examining influences on the policymaking process [35]. This analytical framework can be used retrospectively allowing a comprehensive understanding of the policymaking process and prospectively supporting effective planning and implementation of future policies. Another framework that provided guidance is the stakeholder analysis proposed by Roberts et al. [36], which builds on others such as interest group analysis by Lindblom [37] and an examination of the bureaucratic process and competition between stakeholders by Downs [38]. This framework helped identify relevant groups and individuals, assess their power, resources, and positions on the policy, and their perception and framing of the policy problem [36]. The stakeholder analysis framework was used in combination with the policy triangle framework particularly in the section on actors and the interpretation of results.

The study protocol, interview guide, and consent form for this study were reviewed and approved by the Institutional Review Board at the American University of Beirut prior to data collection.

## Results

Our findings are presented according to the policy triangle framework (Content, Context, Actors, and Process) [35].

### Content

**Figure 2:**
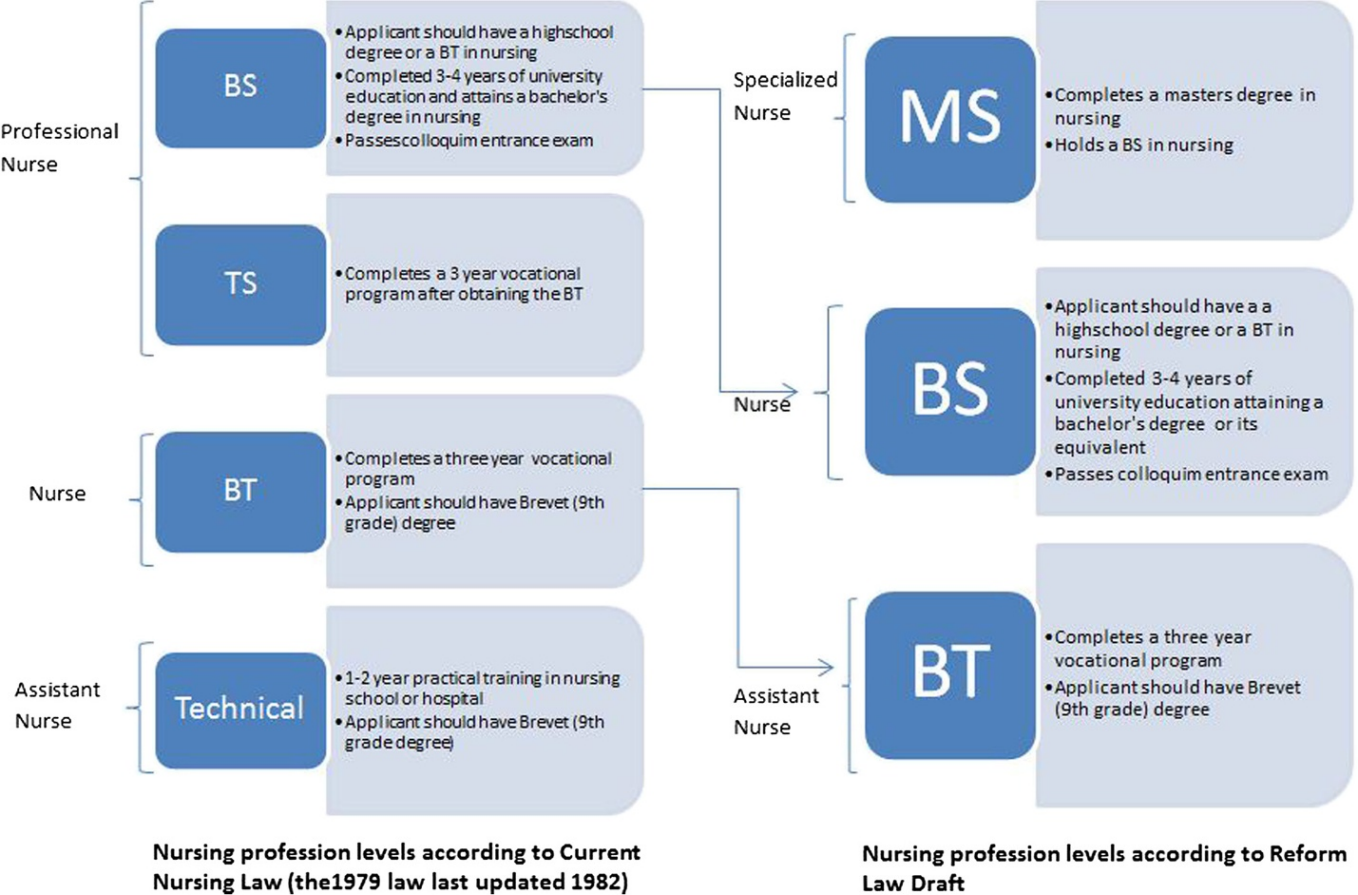
**Nursing levels according to the current and draft nursing practice law.**

The draft nursing practice law’s exact changes required upgrades in each of the educational requirements for nurses and a reorganization of the nursing levels (Figure 2). The draft nursing law classifies the nursing profession into three new categories: i) specialized nurse: a nurse with a Master Degree (MS) in nursing; ii) regular nurse: a nurse with a Bachelor’s degree (BS) in nursing; and iii) assistant nurse: a nurse with a vocational degree in nursing (BT).

Accordingly, the draft law would eliminate the nursing level of students with only 1 to 2 years’ worth of training. It would also stop considering higher vocational degree graduates (TS) as professional nurses and limit regular nurses to those who have a bachelor’s university degree BS. Participants suggested that the draft nursing practice law did not account for the future of the higher vocational degree (TS) as determined by its implementation. Participants also highlighted how the draft law failed to consider the impact of this law on current and future higher vocational degree (TS) nurses though they comprise nearly one third of (33%) the nursing workforce in Lebanon [27] and in some rural underserved areas, they are the only types of nurses available.

Opinions varied on how this law would be implemented; some participants called for the elimination of vocational degrees and others suggested a bridging program from higher vocational degree (TS) to university degree (BS).

“*We cannot abolish TS and BT, we can train them instead*” – A Policymaker

### Context

The proposed draft nursing practice law aimed to modernize a 40-year-old existing law. The analysis of the context revealed various themes, which were categorized under two major themes: promoting factors and barriers.

#### Promoting factors

##### Situation of nursing profession in Lebanon

Participants believed the general situation of the nursing profession in Lebanon to be lacking in many areas. Low salaries, poor working conditions, and unappealing image in Lebanese society were some of the factors plaguing the nursing profession in Lebanon and pushing them towards migration. In fact, studies have demonstrated that nurses choose to migrate out of Lebanon to enjoy a more supportive work environment, autonomy in decision making, career development and promotion, better salary levels, and greater commitment to nursing excellence [31]. Another more recent study among healthcare providers in primary healthcare centers across Lebanon revealed that the top three reasons for quitting are poor salary, better job opportunities outside the country, and lack of professional development [39]. These conditions have led to a shortage in nursing which was considered by most participants to be one of the biggest and most complex problems in Lebanon.

##### Push for incorporation of international standards

Findings revealed that international reports, recommendations, and standards played a significant role in triggering the development of the draft nursing practice law. The formulation of this draft law was prompted by the nursing standards of the WHO and ICN, and the desire to achieve the millennium development goals by 2015 in respect to upgrading nursing education from technical to university level.

##### Disparity in education among nursing schools

An additional factor that demonstrated the need for modifying the nursing practice law is the status of nursing education in Lebanon. All participants acknowledged the need to improve the quality of nursing education by strengthening curricula and encouraging accreditation. Very few nursing schools are accredited by the Ministry of Education which contributes to a discrepancy among various nursing schools. Student admissions into nursing schools are low, yielding an insufficient number of nursing degree holders to satisfy market demands. University hospitals usually employ their graduates (BS holders), leaving technical graduates to public institutions where they are paid less.

##### Disparity in performance of different nursing levels

Participants provided different perspectives as some expressed that there are variations among university degrees and technical degrees in nursing. These differences in performance, compounded by a lack of coordination among educational institutions, led to a perceived decline in the quality of nursing services in the country.

“*It’s a mess between private technical schools, public technical schools, and universities.*” – A Stakeholder

However, few participants perceived vocational graduates as more practical and interactive with patients while considering university graduates more academic. Another mentioned that even though the quality of education may be different, vocational graduates are still delivering quality services, and that vocational and university graduates are delivering the same performance. Some participants stated that even if the higher vocational TS degree education has the same duration as the university, the quality and recognition differs. It was also emphasized that the current system is unfair to vocational graduates as they are paid almost 50% less than university graduates.

#### Barriers

##### Financial hardship of hospitals

One of the factors that impeded the progress of this draft law was the financial situation and hardship that the hospitals were experiencing. The low salaries given to nurses were attributed by the participants to the financial situation especially given the low tariffs and delayed reimbursement by insurers. It was observed that patients usually pay for a doctor’s time, laboratory testing services, bed and medical costs, and prescriptions, but there is no cost of nursing care. This is turn prevents nurses from obtaining their financial rights and makes them feel devalued.

“*The day where patients will begin directly paying for nursing costs on their bills, you will see a radical change in the profession because nurses will become more valued.*” – A Researcher

Participants indicated that this financial situation, in addition to the poor reimbursement for nursing care, made it even more difficult for hospitals to be able to afford the higher salaries of the university level nurses as per the proposed draft nursing law.

##### Disparity in access of students to nursing programs

Participants mentioned that many rural areas in Lebanon lack universities, and technical institutes are the only option they have. This is exacerbated by the fact that technical institutes are more affordable than private universities. There was also a perceived discrepancy in the academic programs and competencies between urban and rural areas in Lebanon. As such, a nursing law that limited the nursing practice to university level graduates would aggravate the shortage and mal-distribution of nurses in Lebanon.

#### Political context

Participants reported that the political environment influenced the policymaking process and added to the delay in the development of this draft. Some participants pointed out that the draft nursing practice law was not high enough on the priority list of the Lebanese government, as it was concerned with other more pressing issues. Also, participants indicated that the poor coordination between the ministries and the unstable political situation in the country hindered the policy process related to this draft law. This may partially explain why 12 years have passed since the introduction of this draft nursing law to Parliament, with no solution as of yet.

In addition, it was mentioned how personal interests influenced policy making process such as favoritism.

“*Under* [the former Minister of Health]*, our efforts were successful due to circumstances: his advisor was married to a nurse… I went to him directly and talked to him… and finally the advisor convinced the minister.*” – A Researcher

Sectarianism, political interests and pressures were also considered to impact policymaking and compromise the transparency of the decision making process.

“*…in Lebanon everything is subject to political and sectarian issues and no one thinks of the health and safety of the citizens and of the harm this can bring to them.*” – A Policymaker

“*Some members of parliament (MP)s agreed on it in the joint committees but those same MPs refused it when it arrived to the general board. They refused because of the favoritism and personal interests.*” – A Policymaker

Though sectarianism did not appear to play a key role in the unfolding of events regarding this particular draft law, participants did mention that some stakeholders (like the vocational schools), were mostly predominated by specific sects (like Shiite sect), which added another layer of political pressure.

Participants explained how economic interests translated into political positions as a result of underlying vested interests policymakers had regarding this law. For example, some participants felt that one of the reasons for the opposition to this law is that some hospitals are owned by politicians and influential people, and many vocational schools are owned by religious associations with their own agendas. Neither group would be willing to pass a law that would require them to relinquish their influence or financial gain. However, few participants showed no concern for political problems in the draft nursing law. They indicated that even though there may be issues among political parties, it is more of a financial problem and less of a political one.

“*You can always blame politics but I’m not sure it’s the right reason. People don’t know how slow the process is.*” *–* A Stakeholder

### Power gradient in the medical field

Another aspect of the formulation of the draft nursing practice law that could have played a role in delaying it was the balance of power and authority among physicians and nurses. Literature indicates that in most Arab countries the medical profession is dominated by males, whereas women dominate the nursing and midwifery professions, which are perceived as “female oriented” and require little or no education, thus falsely creating the poor image of nurses in Lebanon and the region [40]. This gender/power gradient also manifested itself in the legislative authorities as the majority of parliamentarians in the health parliamentary committee that studied the draft nursing practice law, including its president, were male physicians. As for the impact of this draft nursing law on the balance of authority among health professionals, participants had different points of view. Interestingly, though the syndicate of physicians was one of the supporters of this draft law, a few participants predicted that the implementation of this law would make doctors perceive an impingement on their authority and income generation. However, other participants saw the implementation of this law as an opportunity for growth and synergy between the two professions leading to a better use of the physician’s time. Also, some participants believed that it was the responsibility of the Order of Physicians to make sure these two professions do not overlap.

### Actors

**Figure 3:**
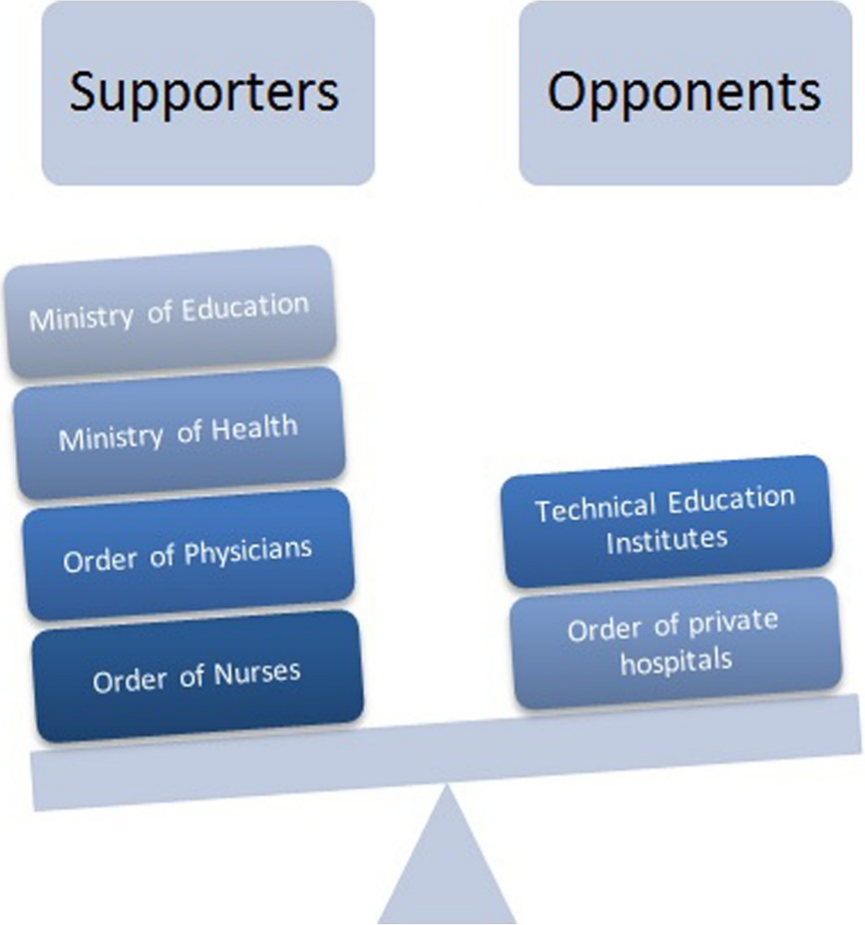
**Stakeholders and their positions regarding the draft nursing practice law.**

With respect to this draft nursing law, the position of the participants in this study ranged from support to opposition (Figure 3). As such, two clusters of actors emerged (supporters and opponents) and they formed issue networks where different individuals and groups are brought together by a common purpose or goal [34]. Taking these networks into account in the analysis would reflect the phenomenon of shared decision making and use of resources to achieve goals [34]. Perception refers to how a problem is characterized, choices are described, and an issue is framed [36]. To this end, stakeholder analysis provided guidance to the analysis of the actors, with a focus on perception as themes relating to the points of controversy of the two clusters or networks of actors were categorized.

The argument for the draft law revolved mainly around three factors: positive impact on nursing profession, enhanced quality of care at the hospital level, and feasibility of switching to the BS degree only.

#### Impact of draft law on nursing profession

Regarding impact on nursing profession, supporters expressed that upgrading the nursing degree to university level would give more value to the nursing profession, which is a main contributor to patients’ health and wellbeing. This would then enhance the image of the nursing profession in Lebanon and lower the rate of migration of nurses thus decreasing the nursing shortage. Supporters criticized the current nursing education system in Lebanon by citing discrepancies in competencies between vocational and university education and ineffectiveness of government examinations in technical schools. All this in their opinion made it necessary to standardize competencies and evaluations. Supporters also claimed that upgrading nursing degrees would lead to improvement in patient health by having better educated nurses and higher quality of services.

On the contrary, opponents expected the nursing shortage to be aggravated due to increased migration rates. Lebanese BS level nurses are sought after by recruitment agencies, and nearly 65% of migrant nurses have a BS degree [8]. Opponents also saw that the law would decrease the stature and scope of practice of vocational graduates, which would lead to decreased enrollment in their programs.

“*We have a shortage of nurses and this new law will increase it. No one will go to a vocational school to become an ‘assistant’ nurse.*” – A Stakeholder

Other participants felt that this draft law was not addressing the real problem, which is the nursing shortage, and that there is a need for more policy-relevant research on the nursing shortage.

“*The study that is needed is the one that informs us on how to increase the number of nurses (how to overcome the shortage) regardless of converting TS to BS. When we have enough BS we can abolish TS and convert them but we are not at this stage yet.*” *–* A Policymaker

Other options according to the opponents included, enhancing the curricula of technical programs, training vocational students, and establishing accreditation of universities and technical institutions to achieve standardization of nursing education.

#### Feasibility of implementing the Nursing Practice Law

When it came to the implementation of this draft law, some supporters felt that it was feasible through creating bridging programs between the vocational (TS) degree and the BS degree as a transition phase, and the reliance on the Lebanese University (free public university) as a venue where educational programs are offered in addition to offering financial aid to students enrolled in private universities.

However, opponents mentioned several implementation barriers like the unaffordability of the education in private institutions, the poor access of students in rural areas to the Lebanese University, and the weak capacity of this university to host enough nursing students to respond to market needs. As such, they predicted that the draft law would decrease enrollment rates and exacerbate the nursing shortage. Meanwhile, technical institutes are available in many areas in Lebanon and provide affordable and accessible nursing education.

“*…technical institutes shouldn’t be closed but instead the quality of their education should be enhanced since the presence of these technical institutes is crucial especially in rural areas where there are no universities and where there is a great need for nurses.*” – A Researcher

Regarding cost considerations, supporters believed that higher education would benefit hospitals by helping them conform to accreditation standards. Better healthcare quality was indicated by some to improve efficiency, enhance patient outcomes, and reduce costs. The syndicate of hospitals countered this argument by claiming that they could not afford to increase the salaries of nurses to the levels required by higher degrees due to the current financial difficulties already observed in trying to pay salaries today. Even though enhancing levels of education would enhance quality of care, thus increasing wages and the cost on patients as well.

Supporters criticized the opponents by claiming that most technical institutes were open for political reasons and are not needs-based, and that their opposition to the draft law was rooted in financial reasons, as they did not want their degrees abolished and to suffer the resultant loss in revenue.

### Process

This section presents the process of the development of the draft nursing practice law and findings are categorized into subthemes including: problem identification, formulation, negotiation, and use of evidence in policy.

#### Problem identification

The draft nursing law was presented to the parliament in 2000 and has been pending for around 13 years. Work on the draft nursing law restarted with the establishment of the ONL in 2002, which was established in response to the need for nurses to have an organized body that enhanced their image and gave them a voice. Hence, the ONL decided to take the lead on re-examining the regulations governing the nursing practice in Lebanon.

Findings revealed that the draft nursing practice law in Lebanon was prompted by the ICN international standards and WHO recommendations concerning organizing the nursing profession. The shortage of nurses in Lebanon and the perceived poor quality of nursing care also played a role in recognizing this issue as a problem and initiating the draft nursing law. One factor that helped get this draft law on the policymaker’s agenda was the fact that the minister of health’s wife was a nurse and the ONL was able to persuade him to champion this draft nursing law.

#### Formulation

One of the participants involved in the development phase of the draft nursing law mentioned that the ONL invited representatives of schools of nursing of the major universities in Lebanon to help in preparing the draft Nursing practice Law. A WHO representative was also present to make sure that the new draft law was in line with and up to the WHO standards of care. Different groups within the ONL were established to work on the competencies, code of ethics and bridging program in the draft nursing law. With respect to the role of research in the development of the content of this draft nursing law, participants cited sources including international reports (WHO, ICN, and World Bank) and information from the ONL. Despite the fact that many said there was poor use of evidence, few participants insisted that this draft nursing law was evidence based, particularly since it was initiated by professionals who understand the importance of research in policymaking processes.

However, local evidence was generally limited to numbers and basic statistical information, and some participants indicated particular types of data that are missing.

“*We have data on the number of nurses graduated but we don’t have information and studies on their career path and their performance*” – A Policymaker

Some participants mentioned utilizing personal contacts midst the absence of reliable sources for obtaining the needed information while questioning its validity.

Even though rigorous local research was limited, some stated that international reports should not be considered as sufficient evidence as they were not specific to the context of Lebanon. Opponents of this draft nursing law believed that, although international standards for nursing are important, examining this profession within the local context should be given equal consideration.

#### Negotiation

After the final draft was prepared, it was submitted to the MOPH for revision and approval. A meeting was held in the MOPH where key pertinent stakeholders were invited to get detailed information and explanations about the draft law and consequently provide their feedback. The majority of the feedback was positive, but representatives of the Order of Private Hospitals and the Vocational and Technical Education expressed various reservations concerning the law. In this meeting, implementation barriers were voiced but not addressed. With respect to the circulation of documents and information pertaining to the draft, some participants indicated that it was not passed to all those concerned.

The dearth of research evidence to support the draft nursing law was mentioned as one of the reasons the committee of justice stopped working on it.

“*There was no evidence presented by the Order of Nurses or Ministry of Health. They only replied verbally to what was presented by the directorate of the technical and vocational education*” – A Policymaker

These conflicts continued on to be reflected in the meetings of the parliamentary committees, and eventually paralyzed their work and halted the progress of this law.

#### Use of evidence in policy

As for the role of research in informing policymaking in Lebanon, some participants said that evidence has a minimal influence due to the overwhelming power of personal and political interest.

“*It depends on a window of opportunity.*” *–* A Researcher

“*Here, politics impedes the use of science*” – A Policymaker

Many participants considered that policy-relevant research in Lebanon is needed in addition to dialogue between policymakers and stakeholders to deliberate about problems and potential solutions.

“*We cannot keep on taking decisions haphazardly.*” – A Stakeholder

**Figure 4:**
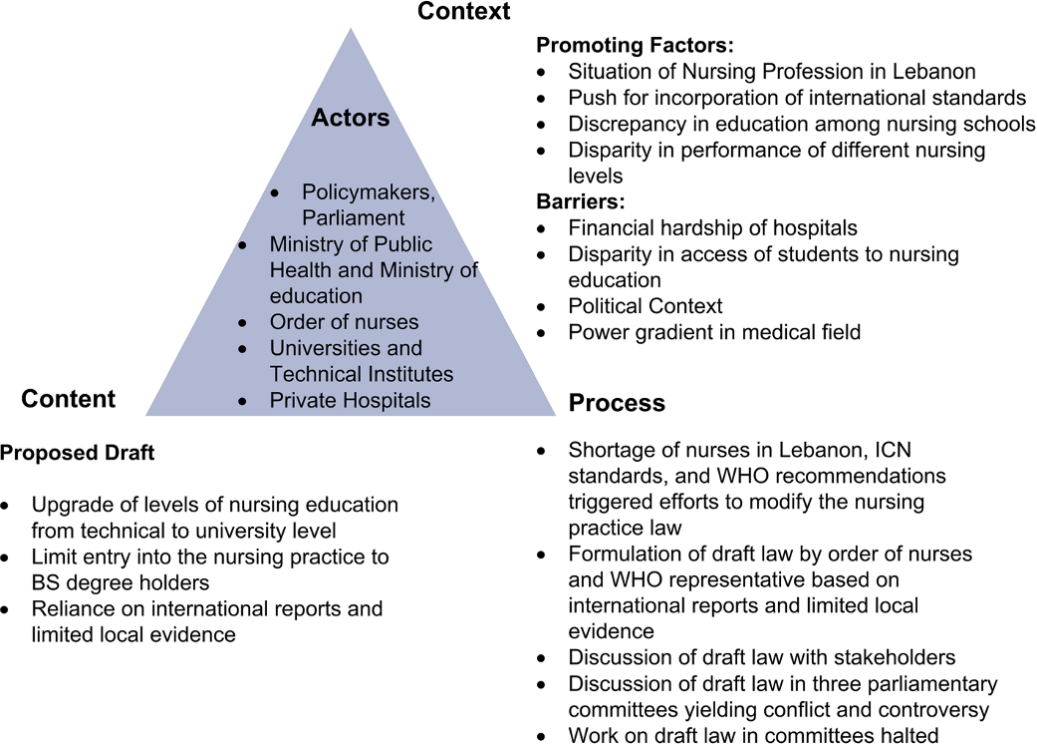
**Content, actors, process, and context in the formulation of the Draft Nursing Practice Law** [35] **.**

Other factors that hinder the use of evidence as mentioned by the participants include the high turnover rate of ministers and policymakers which yields poor commitment to long term strategic planning, and limited research funding by the government, leading to dependence on international financial support. Figure 4 details the content, actors, process and context in regard to the formulation of the Draft Nursing Practice Law.

## Discussion

Study findings shed light on the complexity of the policymaking process and its influencing factors. This policy analysis exercise revealed that successful policy development should take into consideration implementation barriers in the formulation stage. Findings show that the formulation of the draft nursing practice law was hindered by a lack of clarity on the problem, its underlying factors. However, addressing a health systems and policy problem requires working through the underlying problem, options for actions, and implementation issues [41]. The uncertainty that shrouded the problem and relevant solutions in the draft nursing practice law was a result of gaps in knowledge. Such gaps in knowledge included local data on current and future demand and supply of nurses, access to nursing education, and many others. This highlights the need for context-specific evidence.

The study also revealed an absence of a structured decision making approach that utilizes research evidence. Interestingly, despite the availability of some local studies on nurses in Lebanon that could have informed the development of this draft, they were not used in policy development and were disregarded. This implies weakness in translation of knowledge and absence of effective communication between researchers and policymakers. It was observed that policymakers rarely access evidence generated from academic institutions, research centers, or think tanks to address their knowledge needs. Findings suggest the need to establish institutional linkages between policymakers and researchers. Findings also showed that the barriers against the use of evidence in policymaking in Lebanon include political influence, personal interests, lack of research funding, and poor commitment to long-term strategic planning.

Several barriers impeded the progress of the proposed draft nursing law including financial hardships of hospitals, disparity of access of students to nursing programs, and authority gradient in the medical field, in addition to the characteristics of the policy environment (political issues, favoritism, sectarianism, and vested interests). This led to the resistance of various stakeholders involved in this draft nursing law. Stakeholders were given an opportunity to weigh in and voice their concerns but the implementation barriers that they suggested (like the inability to afford higher nursing salaries, or absence of enough universities to generate the required nursing workforce) were never addressed.

Regarding the stakeholders, each had their own sources of power and influence. The main supporters of the nursing practice law were the ONL, MOPH, the Ministry of Education, and the Order of Physicians, who formed a network of actors representing the “State” and medical associations. The power of these groups within the network stemmed from tangible and intangible political resources [36]. Tangible resources included the financial power of the MOPH as an insurer of half the Lebanese population, and the people (HRH) that the two orders represented (nurses and physicians) [36]. Intangible resources included the legitimacy and visibility of these groups [36]. They played the role of stewardship (ministries and orders) and were regarded by other stakeholders and the Lebanese public as a credible source of power and information [36].

As for opponents, the major players were the Order of Private Hospitals and the technical education institutes. As such, this group represented the non-state, private sector and in particular “economic groups” which were industries affected by the health policy [36]. The tangible sources of power of these groups were their ownership of major organizations (hospitals and vocational schools) mobilizing thousands of people and millions of dollars in equity [36]. Private hospitals in Lebanon were almost mainly responsible for secondary healthcare delivery in Lebanon, and vocational schools were responsible for graduating over half of the nursing workforce [26, 28]. As for the intangible sources of political power, these groups had valuable information and knowledge on the problem and options as they were on the implementing end of this policy which gave them legitimacy [36]. Also, they had access to key decision makers and power holders as participants stated some politicians owned hospitals or are close to hospital/vocational school owners [36]. Accordingly, there was no particular interest group powerful and organized enough to push its point of view into formal approval.

In addition, the particularities of the Lebanese political system played a role in delaying this draft law. In Lebanon, a law cannot pass until the majority of the parliament, or members of the parliamentary committee vote for it. Members of Parliament and committee members represent majorities and minorities in the Lebanese political scene. This dispersal of power and mode of decision making gave more attention to smaller interest groups than in legislatures with winner-take-all single member constituencies [36]. Accordingly, a majority was not achieved due to constant conflicts and power struggles. Although all actors agreed on the need to improve the nursing profession in Lebanon, there were many differences as to how it should be done.

Moreover, equity played a major role in the implementation considerations of this draft law. Although equitable and sustainable development had long been a goal of the international and local community, little has trickled down into actions. Disparity in quality of education between nursing schools, disparity in performance of nursing levels, disparity of working conditions across regions and nursing levels, and disparity in geographical and financial access to nursing education are only a few equity problems plaguing the nursing workforce in Lebanon. As such, although equity is well recognized as a focal point for action, it is not being implemented, which highlights the role of other factors specific to Lebanon such as favoritism, sectarianism, and corruption in directing decision making.

This case study also demonstrated the difficulty in the use of global evidence and international recommendations in a local setting without accounting for local applicability. In the development of the draft nursing law, recommendations from international reports were applied as is, with little or no regard to contextual factors and implementations considerations. This resulted in major conflict, delay, and eventual failure to pass the draft nursing practice law. However, health systems guidance should assist decision making [41], not replace it. Contextual factors, in addition to the pros and cons of the options, should be taken into account before adopting specific health systems guidance [41].

Policy making processes and their influence on health care reform and practice – specifically nursing practice – in Lebanon and the EMR are severely understudied. However, this study confirms what has been revealed by other international and regional research on the barriers to policy making. For example, the review by Innvaer et al. [42] reported that some of the most common barriers to evidence-informed policymaking included the lack of timeliness and relevance of research, power and budget struggles, and political instability or high turnover of policymaking staff. Also, a regional study exploring researchers’ views on the use of health systems and policy research evidence revealed that practical constraints to implementation, political interests and sensitivities, and a general non-receptive policy environment hindered the use of evidence and therefore the effectiveness of policy making in Lebanon [6]. The unstructured process for decision making was also revealed by other regional studies [43]. Another policy analysis exercise conducted in Lebanon on the voluntary health insurance policy also revealed the absence of a structured process for policymaking as the policy was formulated and implemented without the use of evidence [44]. Another 2012 study looking at the views of policymakers from 10 countries (Algeria, Bahrain, Jordan, Lebanon Oman, Pakistan, Palestine, Sudan, Tunisia, and Yemen) on the use of health systems evidence, also revealed how political forces are seen as barriers to health policy making processes in general, in addition to the lack of funding and investment in the field of health care and health care research [45].

### Strengths and limitations

Our study has five main strengths. First, to our knowledge, it is one of the very first country case studies conducted in the EMR to closely investigate the policymaking process of a policy that has not yet been ratified. Second, the policy triangle framework, in combination with the stakeholder analysis framework that was used for analysis, helped build a comprehensive understanding of the draft nursing law by identifying its content and objectives, the actors involved in this draft nursing law, the process of policy initiation and formulation, and the context within which the draft law was developed. Third, we interviewed all the key people who were involved in the development of the draft nursing law. This is particularly important in light of the limited documentation. Fourth, the data analysis was conducted through triangulation of data by having two independent reviewers (RH and NA). Fifth, following the interviews, a panel discussion was conducted to validate the results and cross-check the information thereby enhancing the credibility of the study.

Several limitations are also acknowledged, the first of which is temporality and recall bias. Participants might not have been able to correctly recall the events that occurred during the formulation and communication phase of the draft nursing law which spanned over a decade. Some of the information as to the history and process of the draft nursing law was contradicting among several participants, creating difficulties in determining the most accurate and precise information. However, the panel discussion helped in the validation of the process and the accuracy of the information collected. Second, dealing with such a sensitive issue was challenging and effectively engaging the participants was difficult. However, to overcome this obstacle, the team carefully explained to the participants the importance of the case study/research, the neutrality of the researchers, the absence of pre-conceived judgments, and the confidentiality in the way the data was to be dealt with. Further, the research team heavily engaged the ONL in order to garner their support and to ensure that the results of the study would be picked up and fed into the next policy cycle.

A third limitation was the positionality of the researchers. The lead researcher was heavily involved within this field (health policy, knowledge translation, health human resources) and had contributed substantially to the literature on this topic as evident by the relatively large number of references attributed to him. This meant the researcher was an insider in the policy environment which could have biased the research [34]. However, the fact that the lead researcher had enough expertise meant he had access to key information and stakeholders to appropriately understand the culture of the study and ask suitable questions [34]. Further, the research team was multidisciplinary as the study was jointly led by researchers from both a public policy and public health track (not exclusively in the nursing or policy field). This meant the team included outsiders who were able to offer a sense of objectivity in their inherent curiosity and unfamiliarity with the field and its stakeholders [34]. The combination of insiders and outsiders in this team allowed for a rich and comprehensive understanding of the policy process [34].

Fourth, another limitation may have been the agenda of the researchers and their focus on the role of evidence in policymaking as a key tenet in the analysis, which could have biased the interpretation of the data. However, the choice of research question and design of the study was not made based on the researchers’ personal agenda, but in response to policymakers, international calls (Beijing, Montreux and Bamako), and well-acknowledged gaps in knowledge translation and health policy analysis [2–6].

Fifth, as for the application of the two policy frameworks in this policy analysis case study, which is a first in the region, they provided guidance as to how to breakdown the policy development process, identify relevant contextual factors and analyze the stakeholders regarding distribution of power and influence, and problem framing. However, these frameworks (policy triangle, stakeholder analysis) come from the developed/western countries and have not been adapted to suit the political environment in the EMR. As such, interpreting our findings within the dimensions of these frameworks required a degree of adaptation and contextualization. Further, this particular policy analysis deals with a policy that was never passed but stagnated at the phase of development and, as such, the implementation phase in process category was not addressed.

Sixth, from the time of the initial drafting of this case study there might be additional updates regarding the policymaking process. Thus, the information in this study is limited to the date of the end of data collection, which is June 2013.

### Implications for policy and research

Study findings suggest that there are certain steps to be taken before moving on to changing the law governing nursing practice. These steps include standardizing the nursing education programs (university and vocational) and examinations to reflect the competencies required and conducting studies on the best context-specific alternative to the current nursing care model. Accordingly, this study triggers multiple research questions that need to be answered before reaching a best-fit policy change at the level of the nursing education and practice: i) What is the current and projected demand and supply for HRH and nursing workforce in Lebanon? ii) How does the quality of care and performance differ between different nursing levels? iii) What are the different nursing care delivery models followed within the Lebanese hospitals? And what are the educational competencies needed for every model? iv) What would be the optimal nursing care model that can raise the quality of nursing care within the context and resources available while decreasing the nursing shortage? v) What would a collaborative nursing education system look like in Lebanon and how can it be regulated and governed?

Further, it is worth noting that the draft nursing practice law only addressed one aspect of the nursing profession, namely education and competencies, without touching upon other issues. For example, despite the presence of local evidence on the issue, the draft nursing law did not tackle the working conditions of nurses. This indicates the need for a comprehensive nursing law that governs the profession.

Despite the stagnation of the draft law, a recent decision was taken to reduce the years of study of the higher vocational degree (TS) from 3 to 2 years. This caused problems in providing nursing practice licenses and currently the ONL is trying to mitigate this issue using temporary solutions through providing a proposal to the Ministry of Justice. This decision has only increased the discrepancies in the nursing workforce and makes it even more critical to work on advancing a comprehensive draft law.

In addition to grounding the draft law with evidence and answering information gaps, the formulation and negotiation strategies of the law should be changed. The law, its purpose, and implications should be drafted and framed during negotiations in “win-win” terms rather than “win-lose” terms [36]. Moreover, there are some areas where achieving consensus is not possible, and the other party will always look out for their own interests. Some value-dividing conflicts are inevitable [36]. For example, hospitals would have difficulty in increasing nursing salaries and technical institutions would never support an option that will make them lose business. In such cases, a legislative authority can intervene and enforce the decision if it is well grounded in evidence and suitable to the local context. To get through these conflicts, parties should first acknowledge the implementation barriers, express openness to addressing them, and agree to “principle-based negotiations” where a set of principles/values are established at the start of discussions to guide action and resolve disagreements [36]. Interestingly, and based on the experience of the draft nursing practice law, the ONL is revisiting the content of this draft law including the framing of the problem and the proposed solution. For instance, current efforts led by the ONL are focused on retaining the TS program to ensure a good representation in the nursing workforce taking into consideration socioeconomic and geographic factors.

Findings revealed the risk involved in relying blindly on international recommendations and using them without accounting for implementation barriers, contextual factors, and local applicability. When selecting a policy option, an assessment of the key features of a health system that can influence decision making, including governance, financial and delivery arrangements should be conducted [41]. One way to achieve this is to draw on global guidance and context-specific data to develop a policy brief that contextualizes the problem, solutions, and implementation considerations [41]. Needs, availability of resources, costs, modifying factors, and values should be assessed locally with international support to make guidelines applicable [46]. The local adaptation process should be transparent and systematic, involve stakeholders, report influencing factors, and modify guidelines accordingly [46]. Findings suggest the need for capacity building on how to use evidence and apply global guidance within a local context. The study also demonstrates the need for establishing a structured decision making approach that integrates the systematic use of evidence and includes the effective involvement of stakeholders throughout the decision making process from problem identification to policy formulation and implementation. Despite the presence of multiple venues for stakeholders to express their points of view, their feedback did not play a role in shaping the content of the draft nursing practice law.

The significance of the use of evidence in policymaking should be communicated to the public through raising awareness, dissemination of research results, and the media. Public awareness would enhance accountability of policymakers and encourage evidence-informed policymaking. Moreover, the gaps in knowledge revealed by this study, and which hindered informed decision making, suggest the need for better communication between researchers and policymakers.

## Conclusions

Findings shed light on the complex nature of health policymaking, its influencing factors, and the unstructured approach of decision making. This policy analysis case study revealed the barriers to the development and adoption of the draft nursing practice law and to the use of evidence in policymaking. Findings also uncovered the risk involved in the use of international recommendations without the involvement of stakeholders and without accounting for contextual factors, implementation barriers, and local applicability. This exercise presents findings that are useful to legislative bodies and all other stakeholders for strengthening and revising the existing draft nursing law in order to develop an effective alternative that is applicable in Lebanon. This is particularly important since the ONL, under new leadership, is currently making significant efforts in building its capacity in policymaking, in terms of accessing and using evidence, influencing policymaking and legislations, and promoting evidence-based advocacy. In addition, the ONL is engaging and deliberating with other ministries, including the MOPH, Ministry of Education, and Ministry of Labor, about the nursing workforce and other health policy-related matters. Our findings are relevant in the context of Lebanon and the region as policymakers and other stakeholders can learn from this experience when drafting laws. Findings are also relevant at the global level as international organizations can take this case study into account when developing global guidance and recommendations.

## Abbreviations

BS: Bachelor of Science
BT: Baccalaureate technique
EMR: East Mediterranean Region
HRH: Human Resources for Health
ICN: International Council for Nurses
MOPH: Ministry of Public Health
ONL: Order of Nurses in Lebanon
TS: Technique superior
WHO: World Health Organization.
